# Retraction: Territory-wide Chinese cohort of long QT syndrome: random survival forest and Cox analyses

**DOI:** 10.3389/fcvm.2024.1532096

**Published:** 2024-11-28

**Authors:** Frontiers Editorial Office

A Retraction of the Brief Research Report Article Territory-wide Chinese cohort of long QT syndrome: random survival forest and Cox analyses by Tse G, Lee S, Zhou J, Liu T, Wong ICK, Mak C, Mok NS, Jeevaratnam K, Zhang Q, Cheng SH, Wong WT (2021). Front. Cardiovasc. Med. 8:608592. doi: 10.3389/fcvm.2021.608592

Following publication, concerns were raised regarding the ethical approval for this study. An investigation was conducted by Hong Kong Hospital Authority Research Ethics Committee (REC). This committee confirmed that this study did not receive appropriate ethical approval.

An investigation was then conducted in accordance with Frontiers' policies, and it was also confirmed that, contrary to the statement in the article, the study did not receive appropriate ethical approval. Lack of appropriate ethical approval is a breach of Frontiers' guidelines and those of the Committee on Publication Ethics. As such, this article is being retracted.

The retraction was approved by the Chief Editors for Frontiers in Cardiovascular Medicine and the Chief Executive Editor of Frontiers. The authors do not approve of the retraction.

